# *Peganum harmala* Extract Has Antiamoebic Activity to *Acanthamoeba triangularis* Trophozoites and Changes Expression of Autophagy-Related Genes

**DOI:** 10.3390/pathogens10070842

**Published:** 2021-07-04

**Authors:** Rachasak Boonhok, Suthinee Sangkanu, Julalak Chuprom, Mayuna Srisuphanunt, Roghayeh Norouzi, Abolghasem Siyadatpanah, Farzaneh Mirzaei, Watcharapong Mitsuwan, Sueptrakool Wisessombat, Maria de Lourdes Pereira, Mohammed Rahmatullah, Polrat Wilairatana, Christophe Wiart, Lim Chooi Ling, Karma G. Dolma, Veeranoot Nissapatorn

**Affiliations:** 1 Department of Medical Technology, School of Allied Health Sciences, Walailak University, Nakhon Si Thammarat 80160, Thailand; rachasak.bo@mail.wu.ac.th (R.B.); mayuna.sr@wu.ac.th (M.S.); sueptrakool.wi@wu.ac.th (S.W.); 2 Research Excellence Center for Innovation and Health Products (RECIHP), School of Allied Health Sciences, Southeast Asia Water Team (SEA Water Team) and World Union for Herbal Drug Discovery (WUHeDD), Walailak University, Nakhon Si Thammarat 80160, Thailand; zillion.nana@hotmail.com (S.S.); julalak112@gmail.com (J.C.); 3 Department of Pathobiology, Faculty of Veterinary Medicine, University of Tabriz, Tabriz 5166616471, Iran; roghayehnorouzi123@gmail.com; 4 Ferdows School of Paramedical and Health, Birjand University of Medical Sciences, Birjand 9717853577, Iran; asiyadatpanah@yahoo.com; 5 Department Parasitology and Mycology, School of Medicine, Shahid Sadoughi University of Medical Sciences, Yazd 14188-15971, Iran; mirzaei.farzaneh2015@yahoo.com; 6 Akkhraratchakumari Veterinary College and Research Center of Excellence in Innovation of Essential Oil, Walailak University, Nakhon Si Thammarat 80160, Thailand; 1234_k@hotmail.co.th; 7 CICECO-Aveiro Institute of Materials and Department of Medical Sciences, University of Aveiro, 3810-193 Aveiro, Portugal; mlourdespereira@ua.pt; 8 Department of Biotechnology and Genetic Engineering, University of Development Alternative, Lalmatia, Dhaka 1209, Bangladesh; rahamatm@hotmail.com; 9 Department of Clinical Tropical Medicine, Faculty of Tropical Medicine, Mahidol University, Bangkok 10400, Thailand; 10 School of Pharmacy, University of Nottingham Malaysia Campus, Selangor 43500, Malaysia; christophe.wiart@nottingham.edu.my; 11 Division of Applied Biomedical Science and Biotechnology, School of Health Sciences, International Medical University, Kuala Lumpur 57000, Malaysia; chooi_linglim@imu.edu.my; 12 Department of Microbiology, Sikkim Manipal Institute of Medical Sciences (SMIMS), Sikkim 737102, India; kgdolma@outlook.com

**Keywords:** *Peganum harmala*, *Acanthamoeba triangularis*, encystation, RT-PCR, autophagy

## Abstract

*Peganum harmala*, a well-known medicinal plant, has been used for several therapeutic purposes as it contains numerous pharmacological active compounds. Our study reported an anti-parasitic activity of *P. harmala* seed extract against *Acanthamoeba triangularis*. The stress induced by the extract on the surviving trophozoites for *Acanthamoeba* encystation and vacuolization was examined by microscopy, and transcriptional expression of *Acanthamoeba* autophagy-related genes was investigated by quantitative PCR. Our results showed that the surviving trophozoites were not transformed into cysts, and the number of trophozoites with enlarged vacuoles were not significantly different from that of untreated control. Molecular analysis data demonstrated that the mRNA expression of tested *Ac*ATG genes, i.e., ATG3, ATG8b, and ATG16, was at a basal level along the treatment. However, upregulation of *Ac*ATG16 at 24 h post treatment was observed, which may indicate an autophagic activity of this protein in response to the stress. Altogether, these data revealed the anti-*Acanthamoeba* activity of *P. harmala* extract and indicated the association of autophagy mRNA expression and cyst formation under the extract stress, representing a promising plant for future drug development. However, further identification of an active compound and a study of autophagy at the protein level are needed.

## 1. Introduction

Free-living *Acanthamoeba* spp. are widely distributed in the environment, especially in soil and water, and several species of *Acanthamoeba* are known as an opportunistic protozoan in humans [1,2]. *Acanthamoeba* spp. can be transmitted to humans, and several routes of transmission, for example, ocular, nasal, and injured skin, are reported [3,4,5]. The clinical presentation in immunocompromised individuals may be more severe and may present with cutaneous lesions [6], chronic sinusitis [7], and granulomatous amebic encephalitis [8] depending on the site of infection. In healthy individuals, *Acanthamoeba* keratitis (AK) is the most frequent ocular disease caused by this protozoan parasite and has recently been increased among contact lens wearers [9,10]. Regarding *Acanthamoeba* genotypic diversity, the T4 genotype is the major genotype associated with AK [11]. *Acanthamoeba triangularis*, one of the T4 members, has been shown to cause corneal infection in humans [12,13]. According to *Acanthamoeba* life cycle, a metabolically active form is called trophozoite and upon infection; this form can replicate by mitosis while under a stressful condition, transforming into a dormant and resistant stage called cyst which contain double walls [14]. This form is a major obstacle for *Acanthamoeba* treatment, as it is less sensitive to most of available drugs [15,16]. Available drugs for *Acanthamoeba* treatment are relatively limited. Chlorhexidine and polyhexamethylene biguanide (PHMB) are the two most common drugs used for *Acanthamoeba* spp. treatment. However, there was a report showing PHMB resistance in *A. castellanii* clinical isolates [17].

Autophagy is a major intracellular degradative process responsible for degradation of damaged organelles, as well as large protein aggregates [18,19], including intracellular pathogens [20,21]. The autophagic cargo is enclosed in a double-membrane structure, autophagosome, which is further fused with lysosome for degradation. A defect of autophagic mechanism has been related to many diseases in humans such as neurodegenerative diseases [22], cardiomyopathies [23], and non-alcoholic fatty liver disease [24], among others. The autophagosome biogenesis is involved with several autophagy-related (ATG) genes. In yeast and mammals, more than 30 ATG genes have been characterized [18], while in the free-living amoebae including *Acanthamoeba* spp., a partial list of ATG genesis conserved and some have been identified [25,26,27,28]. On the basis of the evidence thus far, a role for *Acanthamoeba* autophagy has not been clearly demonstrated. Some ATG genes have been partially characterized and reported to be associated with the *Acanthamoeba* encystation [25,28,29,30]. Thus, further characterization of the autophagy function, physiological change of autophagy at transcript and protein level in response to stress, and autophagy collaboration with other cellular pathways in relation to encystation are of interest.

In this study, we tested an anti-*Acanthamoeba* activity of *Peganum harmala* seed extract and investigated an autophagic response of surviving *Acanthamoeba* trophozoites in response to stress induced by the extract as well as evaluation of their ability in cyst transformation. The *P. harmala* seed extract demonstrated a significant amoebicidal activity against *A. triangularis* trophozoite. Morphological changes of the amoeba upon treatment were demonstrated by scanning electron microscopy. Along with the amoebicidal activity, the encystation of surviving *Acanthamoeba* trophozoites under microscope and the transcriptional expression of autophagy genes were investigated. Analysis of autophagy mRNA expression was included as an indication of encystation under the extract stress. This raises another point to be taken into consideration, in addition to the cidal activity of the plant extract. Understanding of molecular mechanism in response to a stress further guides a cyst transformation and a potential risk of plant used for the treatment, which gives us insight into the future of drug development.

## 2. Results

### 2.1. Anti-Acanthamoeba Activity of Peganum harmala Seed Extract

**Figure 1:**
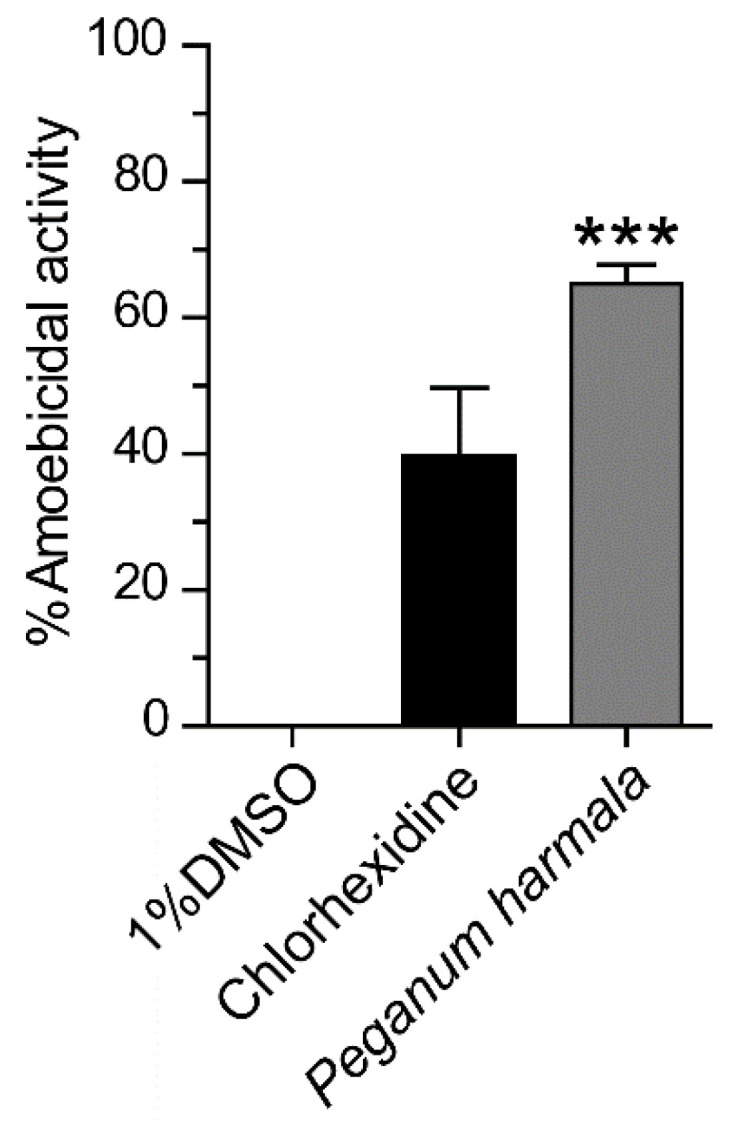
Evaluation of amoebicidal activity of *Peganum harmala* seed extract against *A. triangularis* trophozoites. *Acanthamoeba* trophozoites were treated with the extract for 24 h at a final concentration of 1 mg/mL. Chlorhexidine of 5 µg/mL and 1% DMSO were included as positive and negative controls, respectively. The parasite was stained with PrestoBlue^®^ dye, and the parasite viability was analyzed. The experiment was performed in triplicate with 3 independent experiments. Bar graphs show mean ± SEM. *** *p* value < 0.001.

*Peganum harmala* seed extract was prepared at a final concentration of 1 mg/mL and tested for an anti-*Acanthamoeba* activity against *A. triangularis* trophozoites. The percentage of cidal activity was approximately 65%, and the statistical analysis data showed that it significantly killed the *Acanthamoeba* trophozoite compared to 1% DMSO control (Figure 1).

### 2.2. Determination of IC_50_ of Peganum harmala Extract and Its Morphological Effects

**Figure 2:**
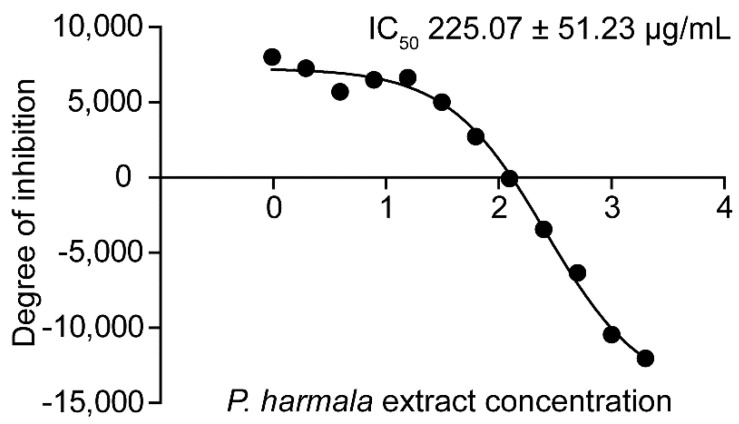
Identification of *P. harmala* extract IC_50_. The *A. triangularis* trophozoites were treated with *P. harmala* seed extract for 24 h and its IC_50_ was determined. Representative image of *P. harmala* extract IC_50_ against *A. triangularis* trophozoites. The data were obtained from 3 independent experiments. The mean ± SD of the IC_50_ is presented.

The *P. harmala* extract had an inhibitory activity against the trophozoite with IC_50_ of 225.07 ± 51.23, while the cyst IC_50_ was 513.07 ± 78.74 µg/mL. The IC_50_ representative images of trophozoite and cyst are shown in Figure 2 and Figure S1, respectively.

A morphological change of the amoeba upon exposure to the extract was observed by SEM. Both trophozoite and cyst forms were treated separately with the extract at RT in the dark for 24 h. The amoebae were then harvested and processed for the scanning electron microscopy. In *P. harmala* extract-treated trophozoites, almost the entire surface of the cells was covered by the extract. The cell membrane was ruptured, and the formation of pores in the membrane was clearly seen (Figure S2). The whole cell of dead parasites was entirely flattened on the surface. In *P. harmala* extract-treated cysts, even the amoebicidal activity was not as potent as expected, and SEM images revealed some morphological changes of the cyst wall (Figure S3). Most of the *A. triangularis* cysts were slightly shrunk, pronounced circular or polygonal edges were obviously seen, and a venation was no longer present on the wall in the extract-treated condition. However, focusing on the membrane pore, we found no different in terms of size and number compared with untreated control.

### 2.3. Drug Combination

The concentration of chlorhexidine and *P. harmala* extract for drug combination assay was varied based on their MICs. The extract and chlorhexidine MICs were 512 and 16 µg/mL, respectively. In drug combination assay, at maximum concentrations of the extract (MIC 512 µg/mL) and chlorhexidine (MIC 16 µg/mL), the percentage viability of trophozoites was in a range of 4–7%. Upon reduction of chlorhexidine concentration to 8 µg/mL in combination with different concentrations of the extract, the percentage viability was increased to 21–36%, but their percentages were similar to that of chlorhexidine-treated condition alone, at 21%. At lower concentrations of chlorhexidine (4, 2, 1 µg/mL), a similar result pattern was observed, and their percentage viability was at a comparable level to that of the drug alone. On the other hand, in the combination of the extract concentration of 512 µg/mL with varying chlorhexidine concentrations, the percentage viability was also in the range of 4–7%. After the reduction of the extract to 256 µg/mL, the percentage viability was increased to 21–34%; however, their percentage was close to the extract-treated condition alone, at 28%. When the extract concentration was reduced to 128, 64, and 32 µg/mL, the set of percentage viability was at a comparable level to the drug-treated condition alone. The mean ± SD of the parasite viability is shown in Table S1. However, the data showed that there was no synergistic, additive, or antagonistic effects in any combinations against *A. triangularis* trophozoites.

### 2.4. Analysis of Cyst Formation and Vacuolization

**Figure 3:**
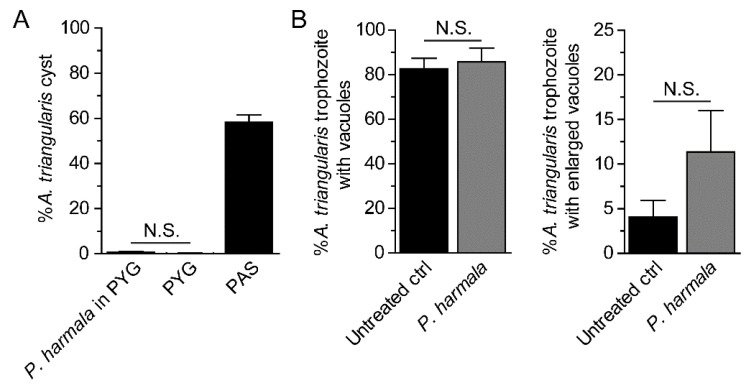
Analysis of cyst formation and vacuolization on surviving trophozoites. The surviving trophozoites were further analyzed for cyst formation and vacuolization after treatment with the *P. harmala* extract at a concentration of 225 µg/mL. (**A**) The percentage of cysts under the extract treatment. Trophozoites cultured in PYG and PAS media alone were included as negative and positive controls for encystation, respectively. (**B**) Surviving trophozoites of at least 100 cells per condition were examined for vacuole formation. Percentages of trophozoites containing vacuoles (**left**) and trophozoites with enlarged vacuole (diameter ≥ 5 µm) (**right**) were analyzed. The data were obtained from 3 independent experiments. Bar graphs show mean ± SD. N.S., not significant.

In *P. harmala* extract-treated condition, the cyst number was at a basal level similar to that of PYG media alone (Figure 3A). The surviving amoebae remained in trophozoite stage. In addition, vacuole formation or vacuolization within the surviving trophozoites of at least 100 trophozoites were analyzed. The trophozoite containing vacuoles, regardless of their size, was first counted. To gain more information upon the treatment, we analyzed the vacuole of diameter ≥ 5 µm, considered as an enlarged vacuole, and the trophozoite with at least 1 enlarged vacuole was counted as 1. The results showed that vacuoles were clearly seen in most of the trophozoites, at 80–90%, and a number of trophozoite containing vacuoles between these two conditions was not significantly different (Figure 3B, left). However, the number of trophozoites containing enlarged vacuole was slightly increased, but it was not significantly different to that of the untreated control (Figure 3B, right). The representative images were shown in Figure S4.

### 2.5. Transcriptional Expression of Autophagy-Related Genes after Peganum harmala Extract Treatment

**Figure 4:**
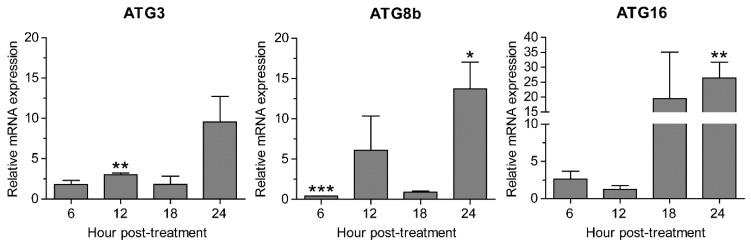
Transcriptional expression of autophagy-related genes. *A. triangularis* trophozoites were cultured in PYG medium in the presence or absence of the extract and incubated for 24 h. The relative changes in mRNA levels of the ATG3, ATG8b, and ATG16 genes were examined every 6 h using quantitative PCR. The level of indicated transcripts from each time point was expressed as a relative mRNA expression. 18S rRNA was used as an internal normalization gene. The expression level was compared to that of time zero, defined as 1. The data were obtained from 3 independent experiments. Bar graphs show mean ± SEM. * *p* < 0.05; ** *p* < 0.01; *** *p* < 0.001.

**Figure 5:**
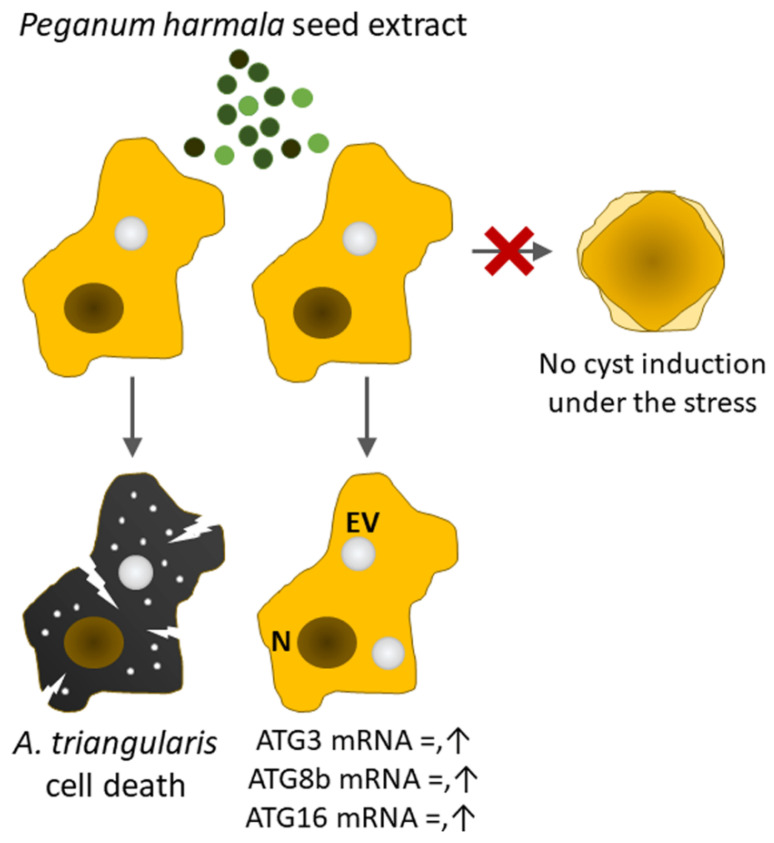
*A. triangularis* response to *P. harmala* seed extract. *P. harmala* extract at concentration of 225 µg/mL was used for the amoeba treatment and approximately 50% of *Acanthamoeba* trophozoites were eliminated. Morphological changes of cell membrane including crack and porous formation that lead to parasite death were examined by SEM. The surviving trophozoites were not transformed to cyst under the stress-induced by the extract. The change of mRNA expression of encystation-related genes was determined. The mRNA expression level of tested ATG genes i.e., *Ac*ATG3, *Ac*ATG8b, *Ac*ATG16 was slightly changed and close to a basal level. However, at later time point, their expression was slightly increased and *Ac*ATG16 was significantly up-regulated at 24 h after treatment. The mRNA expression of cellulose synthase and serine proteinase was fluctuated and demonstrated a similar profile. Their expression was sharply increased at 6 h after treatment, remained at a basal level at 12 and 18 h, and increased again at 24 h. EV, enlarged vacuole; N, nucleus.

Validation of PCR primers targeting ATG3, ATG8b, ATG16, CS, SP, and 18S rRNA genes was first conducted against *A. triangularis* DNA. The target genes were successfully amplified, and a single band of each PCR reaction was clearly demonstrated on the agarose gel (Figure S5). These amplicons were subsequently sent for sequencing to confirm a specific amplification, and the DNA sequencing results are shown in Table S3. Quantitative PCR was then performed using these primers in order to investigate mRNA expression level of the target genes in samples of *P. harmala* extract-treated *A. triangularis* trophozoites of different time points. Regarding the tested autophagy-related genes, our qPCR results showed that after being exposed to the *P. harmala* extract, mRNA expression of *Ac*ATG genes, i.e., ATG3, ATG8b, and ATG16, was slightly changed along the treatment. However, at 24 h after the treatment, the significant increase of ATG8b and ATG16 was observed (Figure 4). The overall results indicate an impaired mRNA expression of these autophagy-related genes. We also observed other encystation-related genes, i.e., cellulose synthase and serine proteinase. Surprisingly, mRNA expression of both genes was markedly increased at 6 h, declined at 12 and 18 h, and then peaked again at 24 h after treatment (Figure S6). Altogether, *A. triangularis* trophozoite response to *P. harmala* seed extract is depicted in Figure 5.

## 3. Discussion

*Acanthamoeba* infection has increased annually, especially in healthy individuals who wear contact lenses [10,31,32]. Together with drug resistance reports [17,33] and a limited number of anti-*Acanthamoeba* drugs in the market [2,34], the identification of new medicinal plants for therapeutic purposes of *Acanthamoeba* infection is needed [35,36]. In addition, drug combination therapy is another strategy to delay drug resistance in infectious diseases [37,38]. To date, several plant extracts have been reported concerning their amoebicidal activity, such as *Curcuma longa* L. [36,39], *Artemisia argyi* [40], *Citrus* spp. [36], *Amomum uliginosum* [36], and *Caesalpinia pulcherrima* [41], among others. Drug combination study of the plant extract with standard anti-*Acanthamoeba* drugs may provide another milestone of information for further *Acanthamoeba* infection therapy. 

*Peganum harmala* has been widely used in diverse therapeutic purposes [42] as it contains several pharmacological activities, for example, anti-cancer [43], anti-oxidant [44], anti-inflammation [45], and anti-microbial activities, which included anti-viral [46], anti-bacterial [45], anti-fungal [47], and anti-parasitic [48,49] effects. Our study discovered the anti-*Acanthamoeba* activity of the *P. harmala* seed extract against *A. triangularis*. As expected, the trophozoite is more sensitive to the extract than cyst due to its cell wall structure. Its IC_50_ was 225 µg/mL, approximately two times lower than that of cyst IC_50_. However, the parasite susceptibility varied depending on the *Acanthamoeba* spp. and the method for the extract preparation [50]. We also observed the effect of *P. harmala* seed extract on the *Acanthamoeba* trophozoites and cysts by SEM. The exposed trophozoites demonstrated pores and cracks on the parasite membrane. The loss of membrane integrity can lead to a leakage in the cytoplasmic content and cell death. This finding is in agreement with a previous study of Irene Heredero-Bermejo and her colleagues in 2020, describing how membrane alteration led to the *Acanthamoeba* cell death [51]. In addition, the presence of pores on the membrane after being exposed to the extract may indicate the amoebic cell death by necrosis [52]. Interestingly, in cyst, a swelling of the pronounced edge, round shape, and smaller size were clearly seen. This morphological change may be a sign of cell death, or it may be a protective mechanism of the parasite under stress conditions. To support this finding, an earlier study by Tisha Lazuana and her colleagues revealed that a perfectly round shape and a decreased size of cyst were observed after exposure to cellulose enzyme and disinfectant solution, and this led to *Acanthamoeba* cell death [53]. However, the characterization of cell death mechanism by *P. harmala* extract is of interest to further verify this association before any conclusion could be made. Our drug combination data of the *P. harmala* extract with chlorhexidine did not show any synergistic effect in any combination below its MIC. Further identification of active compounds and investigation on drug combination will allow us to come to more conclusive findings for future drug development purposes.

In this study, we interested in *Acanthamoeba* autophagy and its association with the stress induced by the *P. harmala* extract. We first observed cyst formation and vacuolization on the surviving trophozoites after treatment with the extract at IC_50_. Our data showed that there was no induction of cyst formation upon the treatment with the extract. This may indicate a specific stress-induced encystation in *Acanthamoeba* spp. and may be a good sign for future use of the plant extract. As mentioned earlier, autophagy is an intracellular degradative process involved with vesicle trafficking, and it can be induced by various stresses. Induction of autophagy leads to an increase of intracellular vacuole [54,55]. To our results, the percentage of surviving trophozoites containing enlarged vacuole was slightly increased upon treatment. Expansion of the vacuole size may indicate a physiological change within the trophozoite in response to the stress induced by the *P. harmala* extract, and this change may be associated with basal autophagy in *Acanthamoeba*. The result was slightly different from our recent published data [56], that upon *Cassia angustifolia* leaf extract treatment, the percentage of the surviving parasite containing the enlarged vacuole was not increased. However, either *P. harmala* or *C. angustifolia* extract did not induce cyst formation on the surviving amoebae. These data indicate the ability of the surviving trophozoites to cope with the stress induced by the extract.

*Acanthamoeba* encystation is a mechanism in which it transforms to cyst. This inactive form is more resistant to environmental stress than trophozoite, becoming a barrier for *Acanthamoeba* treatment. Several pathways are involved with *Acanthamoeba* encystation, including autophagy [27,57,58]. A number of autophagy-related genes have been reported to be involved with encystation, for example, ATG3 [29], ATG8b [30], ATG16 [28], etc. ATG8 was the first *Acanthamoeba* ATG gene to be characterized in this parasite [26]. Data from the literature demonstrated that *A. castellanii* Atg8 protein was highly expressed during encystation and dispersed in cytosol of *Acanthamoeba* trophozoites, while in cyst, forming as puncta structures was seen. The Atg8 positive membrane was later identified as an autophagosome because of its co-localization with lysosome, visualized by LysoTracker staining [26]. *Ac*Atg8b was later identified as another isoform of Atg8, and it was found to be highly expressed during encystation. In addition, their RNA interference data revealed that decreased expression of *Ac*ATG8b mRNA significantly reduced *Acanthamoeba* encystation [30], which indicates the importance of this isoform. *Ac*Atg3 protein, a part of the Atg8 conjugation system, was further characterized in *A. castellanii*. Molecular analysis data by Eun-Kyung Moon and his colleagues in 2011 showed that *Ac*ATG3 mRNA expression level was not changed during the encystation. However, the data by microscopic examination showed that the formation of mature cyst was significantly reduced in Atg3-depleted cells [29]. *Ac*Atg16 protein was later identified and was highly expressed during the *A. castellanii* encystation [28]. Their immunofluorescence analysis data showed that *Ac*Atg16 protein was associated with vesicular structure and partially colocalized with autophagolysosome. The decreased of this protein inhibited the formation of autophagosomes and further affected the cyst formation efficiency [28]. However, our microscopic data showed that the surviving amoebae were not transformed into cyst. Thus, it is interesting to see the expression of autophagy mRNAs in these amoebae. Our results showed that *Acanthamoeba* autophagy responded rapidly to *P. harmala* seed extract as shown by the change of transcriptional expression of ATG mRNAs at 6 h after treatment. The quick response to cellular stress is a key characteristic of autophagy as is seen in other organisms [19,59]. As expected, *Ac*ATG3 mRNA expression was consistent along the treatment. However, *Ac*ATG8b and *Ac*ATG16 demonstrated a similar pattern as their expression was significantly increased at a later time point. This may indicate their role in basal autophagy or autophagy induction for encystation after 24 h treatment. Due to the decreased of *Ac*ATG8b mRNA expression at 6 h after treatment, in addition to a rapid sensing of this autophagy gene to the stress, this could be due to the extract in indirectly mediating the downregulation of the transcript However, further investigation on its role under stress is of paramount importance. Although the DNA sequencing of ATG8b amplicon from *A. triangularis*, approximately 130 bp, did not match to *A. castellanii* NCBI database, the similar amplicon size was obtained. In addition, our conventional PCR result demonstrated a single band of ATG8b PCR product derived from *A. castellanii* ATG8b primer, and the qPCR data also showed a single melt curve, indicating a single PCR product. It is possible that the primers can specifically bind to a conserved sequence in *A. triangularis*. However, the sequence in between the primers may vary and different from that of *A. castellanii*. The variability of genome sequence among *Acanthamoeba* species was supported by the recent genomic data analysis of *A. triangularis* by Issam Hasni et al. in 2020 [12] in that there was a difference of genome size, number of predicted proteins, and proportion of genes in each category among *Acanthamoeba* species. On the other hand, the increase of *Ac*ATG16 may indicate other functions of this protein under the stress. In our recent study [56], the *A. triangularis* trophozoites treated with *C. angustifolia* extract and the ATG mRNA expression of ATG genes in surviving amoebae was analyzed in a similar manner. A similar pattern of the mRNA expression was observed. At later time point of the treatment, ATG3 and ATG8b were significantly increased, while ATG16 was slightly increased. It is possible that one of the active compounds within the extract may work at this time point and activates the basal autophagy, which may not be associated with encystation. However, further investigations on encystation after 24 h treatment as well as isolation and purification of bioactive molecules are needed to characterize a specific response related to a certain ATG transcript. Regarding Atg protein function in other systems, Atg3, Atg8, and Atg16 proteins normally work together at basal autophagy and for autophagosome biogenesis upon autophagic induction. However, the autophagosome biogenesis requires an increased expression of core Atg proteins. In fact, the association between Atg proteins and *Acanthamoeba* spp. is not well understood. Thus, a set of *Acanthamoeba* autophagy proteins working at a basal level for degradation and autophagy induction for encystation may be different. Therefore, a comprehensive study at protein level would definitely provide more in-depth information on how each Atg protein works in *Acanthamoeba* spp., including its association with ATG mRNA level.

Our study also evaluated the mRNA expression of cellulose synthase (EDCBI66TR) and serine proteinase (EU365404) in surviving trophozoites under *P. harmala* extract stress condition. Cellulose is a major component of *Acanthamoeba* cyst wall [60,61], and cellulose synthase is one of the key enzymes required for cellulose synthesis highly expressed during an early phase of encystation [62]. Moreover, another study demonstrated that small interfering RNA (siRNA) of cellulose synthase downregulated its mRNA expression and inhibited maturation of *A. castellanii* cyst [63]. In our study, the surviving trophozoites were not transformed into cyst under the extract pressure. However, the fluctuation of cellulose synthase mRNA expression was observed. Early response of this gene may indicate another function in addition to cellulose synthesis during encystation. The increase of its expression at a later time point may indicate its activity dealing with the stress or be ready for encystment after 24 h treatment. In line with this finding, a previous study by Eun-Kyung Moon and his colleagues in 2007 reported that approximately 16 genes were upregulated during encystation using differentially expressed gene (DGE) screening by RT-PCR [64]. Genes with high expression level were, for example, xylose isomerase, Na P-type ATPase, and serine proteinase. Many studies have investigated a role of proteinases in *Acanthamoeba* spp., and some proteinases are found to be associated with *Acanthamoeba* pathogenesis [65,66]. Serine proteinase was of interest by Eun-Kyung Moon and his colleagues at that time, and the encystation-mediating serine proteinase (EMSP) was later characterized [67]. Our qPCR data showed that the pattern of the serine proteinase mRNA expression was similar to that of the cellulose synthase, and the increased expression at later time point may indicate other functions of this protein or the surviving trophozoite may undergo encystation.

## 4. Materials and Methods

### 4.1. Plant Collection and Preparation of Extract

A dried medicinal plant of *Peganum harmala* (wild rue) seed, Nitrariaceae family, was obtained from a local market in Azerbaijan. The plant species was identified by the Agricultural Research Center in eastern Azerbaijan. This plant was mechanically powdered using an electrical blender. The extract preparation was modified from Boonyadist Vongsak et al. (2013) [68]. The ethanolic extract was prepared by maceration 370 g of dry powdered plant in 70% ethanol for 3 days at room temperature (RT) and filtrated through a filter paper (Whatman Ltd., Buckinghamshire, UK). Then, the ethanol was removed by a vacuum rotary evaporator. Finally, the dry powder extract was obtained after incubation in 37 °C incubator.

### 4.2. Acanthamoeba Cultivation

*A. triangularis* trophozoite, strain WU19001 [39], was cultured with peptone–yeast extract–glucose (PYG) medium (2% (*w*/*v*) proteose peptone, 0.1% (*w*/*v*) yeast extract, 400 µM CaCl_2_, 4 mM MgSO_4_, 2.5 mM Na_2_HPO_4_, 2.5 mM KH_2_PO_4_, 50 µM (NH_4_)_2_Fe(SO_4_)_2_, 100 mM glucose). The amoeba was maintained at RT in the dark without shaking [69]. The PYG medium in the flask was replaced with fresh medium every 2 days until trophozoite harvesting. To obtain *Acanthamoeba* cyst, the trophozoite was cultured with Page’s saline (PAS) medium [70] for at least 5 days. A ready-to-use PAS powder (HiMedia, Mumbai, India) of 0.403 g was dissolved in 1 L distilled water in which the PAS solution contained NaCl 0.120 g, MgSO_4_·7H_2_O 0.004 g, CaCl_2_·2H_2_O 0.004 g, Na_2_HPO_4_ 0.142 g, and KH_2_PO_4_ 0.136 g. Then, the PAS was supplemented with 10% glucose. Cyst was then harvested by centrifugation.

### 4.3. Determination of Anti-Acanthamoeba Activity

A stock concentration of the plant extract was prepared at 100 mg/mL with 100% DMSO. The 96-well black plate (SPL Life Sciences, Seoul, Korea) was used for the amoebicidal activity testing and the final concentration of the extract was at 1 mg/mL [56]. For *Acanthamoeba* trophozoite harvesting, the parasite suspension was centrifuged, and the supernatant was discarded. The PAS was then added to the cell pellet and gently resuspended. This procedure allows for the removal of the old PYG medium and keeps the parasite in a fresh PYG medium before starting the experiment. For cysts, after washing, the amoeba was prepared in PAS supplemented with 10% glucose to create a nutrient-depleted condition to prevent *Acanthamoeba* excystation. Then, the amoebae were counted in a hemocytometer. The *Acanthamoeba* trophozoites were added to the final cell number of 2 × 10^4^ cells per well. The parasite was treated with 5 µg/mL chlorhexidine as a positive control and 1% DMSO as a negative control. To prevent medium evaporation, all edge wells were filled with PAS media. After 24 h treatment, the PrestoBlue^®^ dye (Invitrogen, Waltham, MA, USA), a new reagent based on resazurin to assess cell viability and cytotoxicity, was used for parasite staining at the final dilution of 1:20. The dye was incubated with the parasite in 37 °C incubator for 30 min. Then, the fluorescence signal was quantified by microplate reader (BioTek SynergyTMMX microplate reader, Winooski, VT, USA) at excitation/emission wavelength of 535/615 nm. The parasite viability was further calculated by the Prism 5 software (GraphPad Software, CA, USA). DMSO-treated condition was set to 100% cell viability. Then, calculation of the percentage of anti-*Acanthamoeba* activity was performed using a formula of percentage of amoebicidal activity = 100% cell viability. All experiments were conducted in triplicate with 3 independent tests.

### 4.4. Determination of Inhibitory Concentration 50 (IC_50_)

IC_50_ of *P. harmala* extract against *Acanthamoeba* trophozoites and cysts was further identified. The protocol was modified from Faiza Amber Siddiqui et al. (2020) [71]. In brief, the assay was performed in a 96-well black plate (SPL Life Sciences, Seoul, Korea). The plant extract was prepared with twofold serial dilution. Then, 100 µL of 2 × 10^4^ cells/mL of trophozoites and cysts were added to each well. Untreated parasite and medium alone were included as controls. Plates were incubated at RT in the dark for 24 h. Then, the PrestoBlue^®^ reagent was added into the plate, incubated at 37 °C for 30 min, and a fluorescence signal was quantified by microplate reader (BioTek SynergyTMMX microplate reader, Winooski, VT, USA) at excitation/emission wavelengths of 535/615 nm. The fluorescence signal was normalized by medium alone, and the IC_50_ was calculated using the Prism 5 software (GraphPad Software, CA, USA). All experiments were conducted in triplicate with 3 independent experiments.

### 4.5. Analysis of Cyst Formation and Vacuolization

An IC_50_ concentration of *P. harmala* seed extract on surviving trophozoites was used against trophozoites to evaluate the stress. Then, cyst formation and vacuolization were investigated. The trophozoite culture in PAS and PYG media were used as positive and negative controls for cyst induction, respectively. In brief, the 96-well plate containing *Acanthamoeba* trophozoites was treated with a *P. harmala* extract at a concentration of 225 µg/mL for 24 h. Then, the surviving trophozoites were assessed for cyst formation and vacuolization. The parasite was stained with Trypan blue, and the viable cells were further analyzed. At least 200 cells per condition were analyzed for cyst formation, and at least 100 surviving trophozoites were examined for vacuolization under a light microscope (Olympus BX41TF, Tokyo, Japan). The trophozoite with vacuole, regardless of its size, and the trophozoite containing enlarged vacuole were evaluated. Enlarged vacuole (EV) was defined as a vacuole with a diameter of ≥5 µm, and the trophozoite containing at least 1 EV was counted as 1. Three independent experiments were performed.

### 4.6. Scanning Electron Microscopy

The effect of *P. harmala* extract on the *Acanthamoeba* trophozoites and cysts was further investigated. The protocol for treatment and sample preparation for SEM was obtained from Suthinee Sangkanu et al. (2021) [72]. Parasites of both forms were treated with the extract separately at the final concentration of 225 and 513 µg/mL, respectively, in a 24-well plate. Two pieces of sterile glass with high affinity of parasite binding, approximately 3 × 3 mm^2^ in size, were dropped into the well. Parasites were gently resuspended and then allowed to sit for 24 h. The parasite-coated glass was fixed with 2.5% glutaraldehyde overnight and dehydrated with a series of graded ethanol (20%, 40%, 60%, 80%, 90%, and 100%). Parasite samples were mounted on aluminum stubs and dried in a critical point dryer, followed by coating with gold particles (Cressington 108 sputter coater, MA, USA). The surface structure of the parasite was then observed under SEM (Gemini, Oberkochen, Germany).

### 4.7. Determination of Minimal Inhibitory Concentration (MIC) and Drug Combination

Drug combination is another strategy for an effective treatment of many infectious diseases [37,73]. Chlorhexidine is an active drug against *Acanthamoeba* spp. We then performed drug combination study, chlorhexidine and *P. harmala* extract, and explored its synergistic efficacy against *A. triangularis* trophozoites. First, the minimum inhibitory concentration (MIC) of the extract and chlorhexidine was identified along with the microtiter broth dilution method [39]. A 96-well clear plate (SPL Life Sciences, Seoul, Korea) was used for the extract preparation. The extract was prepared with PYG medium to obtain twofold serial dilution at the concentrations of 2048, 1024, 512, 256, 128, 64, 32, and 16 µg/mL in a 96-well plate. Then, 100 μL of PYG medium was dispensed into each well in the columns 2 to 8, and 100 μL of the extract from column 1 (2048 μg/mL) was transferred to column 2. The extract was gently mixed, and the dilution was repeated up to column 8. The amoebal inoculum was harvested by centrifugation at 3000 rpm for 10 min, and the amoebal pellet was resuspended in fresh PYG medium. Then, they were counted under inverted microscope and adjusted to a concentration of 2 × 10^5^ cells/mL. Then, 100 µL of inoculum, final cell number of 2 × 10^4^ cells, was added into each well. Thus, the final concentrations of the extract were 1024, 512, 256, 128, 64, 32, 16, and 8 µg/mL. Chlorhexidine was performed in the same manner as the extract with the concentrations of 64, 32, 16, 8, 4, 2, 1, and 0.5 µg/mL. Chlorhexidine and 1% DMSO were included as positive and negative controls, respectively. Plates were incubated at RT for 24 h in the dark box to avoid contamination. Then, cells were stained with Trypan Blue dye for cell viability quantification under a light microscope, Eclipse TE2000-S (Nikon, Tokyo, Japan). The parasite viability was calculated as follows: percentage viability = (mean of the viable parasite/control) × 100. The MIC was defined as the lowest concentration that inhibited > 90% of parasite growth. MIC results of the extract and drug against the trophozoite at 24 h treatment were 512 and 16 µg/mL, respectively. The drug combination study was conducted in a similar manner as MIC identification against trophozoite [72]. The experiment was performed in a 96-well plate with a final volume of 200 µL, and the final cell number was 2 × 10^4^ cells per well in PYG medium. Their MICs were used as a starting concentration. The extract and chlorhexidine were diluted with PYG in a microcentrifuge tube to obtain 4 times their final concentrations of MIC, 1/2 MIC, 1/4 MIC, 1/8 MIC, and 1/16 MIC (512, 256, 128, 64, and 32 µg/mL, respectively). A total volume of 200 µL was made in each well by distributing 50 µL of the extract, 50 µL chlorhexidine, and 100 µL of parasite suspension, final cell number of 2 × 10^4^ cells per well. Plates were incubated at room temperature for 24 h. The parasite viability was then quantified.

### 4.8. Preparation of Total RNA and cDNA Synthesis

*Acanthamoeba* trophozoites of 2 × 10^5^ cells per well were cultured in a 24-well plate. The parasite was treated with the *P. harmala* extract at a final concentration of 225 µg/mL, and the plate was incubated at RT for 24 h. Parasites were harvested at different time points, i.e., 6, 12, 18, and 24 h after treatment. Each time point, the parasite of untreated and *P. harmala* extract-treated cells was harvested and transferred to 1.5 mL Eppendorf tubes. The tube was kept on ice along the process. After centrifugation, the culture medium was discarded and the parasite pellet was mixed with 500 µL of TRI reagent (Molecular Research Center, Cincinnati, OH, USA) to protect parasite RNA [56]. Total RNA extraction was done by RNA extraction kit (Vivantis Technologies, Selangor, Malaysia), and a 100 ng of total mRNA was converted to cDNA using the Viva cDNA synthesis kit (Vivantis Technologies, Selangor, Malaysia) following the manufacturer’s protocol. The cDNA sample was preserved at −20 °C until use.

### 4.9. Validation of PCR Primers

List of primers targeting *Acanthamoeba* genes in this study is indicated in Table S2. The genes of interest were ATG3 (GenBank accession no. GU270859), ATG8b (GenBank accession no. KC524507.1), ATG16 (GenBank accession no. FJ906697), cellulose synthase (CS) (GenBank accession no. EDCBI66TR), and serine proteinase (SP) (GenBank accession no. EU365404), and 18S rRNA was used as a reference gene. The primers were first tested against *A. triangularis* strain WU19001 DNA by conventional PCR [74]. To confirm primer specificity, the PCR product was sent for sequencing (Apical Scientific Sdn. Bhd., Selangor, Malaysia), and the DNA sequence was then analyzed and compared with *A. castellanii* NCBI databases before performing a quantitative PCR.

### 4.10. Analysis of Gene Expression by Quantitative PCR

The transcriptional expression of encystation-related genes on the surviving trophozoites after the *P. harmala* extract treatment was investigated. Even when the role of autophagy is not clearly demonstrated in *Acanthamoeba* spp., a number of the *Acanthamoeba* autophagy-related genes were reported to play a role in encystation [28,29,30]. The protocol was modified from Faiza Amber Siddiqui et al. (2020) [71]. The iTaq Universal SYBR Green Supermix Kit was obtained from Bio-Rad (Bio-rad, Hercules, CA, USA), and the quantitative PCR (qPCR) reaction was prepared according to the manufacturer’s protocol. The 18S rDNA was used as a housekeeping control. In brief, 10 µL of iTaq Universal SYBR Green Supermix, 2X concentration was mixed with 100 ng cDNA, 1 µL of 200 mM F + R primers in PCR tube. The total volume was adjusted with DEPC water up to 20 µL. The software setting of thermal cycler, StepOnePlus Real-time PCR systems (Applied Biosystems, Waltham, MA, USA), was as follows: holding stage 95 °C for 30 s; cycling stage for 40 cycles at 95 °C for 15 s; 60 °C for 60 s; and then melting curve stage at 95 °C for 15 s, 60 °C for 60 s, and 95 °C for 15 s with a temperature increase of 0.3 °C. The average ΔCt (deltaCt) was obtained by the thermal cycler. The ΔΔCt and a relative expression of the mRNA were calculated as follows: the ΔΔCt = [(Ct of treated sample GOI-Ct of treated sample housekeeper) − (Ct of untreated control GOI-Ct of untreated control housekeeper)], where GOI is gene of interest; the relative expression = 2 to the power of (minus X) or 2^−X^ where X is ΔΔCt. The interpretation is if the value > 1 means the expression is increased; <1 means the expression is decreased; and if the value is equal to 1, this means the expression does not change.

### 4.11. Statistical Data Analysis

All data were collected and recorded in Microsoft Excel 2016 (Microsoft Corporation, Washington, DC, USA). The assays were performed in 2–3 technical replicates with 3 independent experiments. The statistical analysis of mean ± SD or ± SEM including a two-tailed unpaired Student’s *t*-test by the Prism 5 software (Graphpad Software, San Diego, CA, USA) was used. *p*-values below 0.05 were considered significant.

## 5. Conclusions

Our study presents an amoebicidal activity of *P. harmala* seed extract and its effects on *A. triangularis*, especially the surviving trophozoites. The investigation of *A. triangularis* autophagy-related gene expression at transcriptional level has been explored to see its association with cyst formation by microscopy, which might be a good indication for future plant extract screening and evaluation of a risk for cyst transformation after being treated with plant extract stress sensing by *A. triangularis* autophagy, with intracellular pathways involved with its encystation being of interest and requiring further studies. This study provides autophagy information of *A. triangularis*, another pathogenic parasite of T4 genotype member, and insights into autophagic mechanism in response to stress conditions. Understanding of this mechanism may be useful for future drug development for *Acanthamoeba* infection.

## Data Availability

Not applicable.

## Supplementary Materials

The following are available online at https://www.mdpi.com/article/10.3390/pathogens10070842/s1, Figure S1: *P. harmala* extract IC_50_ against *A. triangularis* cysts. Figure S2: Scanning electron microscopy imaging of *A. triangularis* trophozoites treated with *P. harmala* extract. Figure S3: Scanning electron microscopy imaging of *A. triangularis* cysts treated with *P. harmala* extract. Figure S4: Representative images of vacuolization in surviving *A. triangularis* trophozoites upon *P. harmala* extract treatment. Figure S5: Gel electrophoresis of PCR product. Figure S6: Transcriptional expression of other encystation-related genes. Table S1: Effect of *Peganum harmala* seed extract in combination with chlorhexidine against *Acanthamoeba triangularis* trophozoite. Table S2: List of primers for quantitative PCR. Table S3: *Acanthamoeba triangularis* DNA sequence by Sanger sequencing.
