# Supplementary material for: Peganum harmala Extract Has Antiamoebic Activity to Acanthamoeba triangularis Trophozoites and Changes Expression of Autophagy-Related Genes
Source: Pathogens. 2021 Jul 4;10(7):842. doi: 10.3390/pathogens10070842 (PMC8308471; doi:10.3390/pathogens10070842)
Supplement: Supplementary file 1 [file pathogens-10-00842-s001.zip › pathogens-1234112-supplementary.pdf]

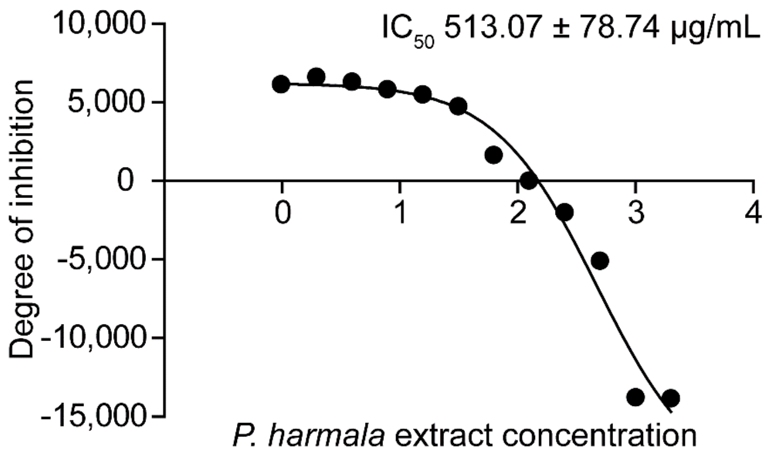

Figure S1. *P. harmala* extract IC_{50} against *A. triangularis* cysts. Cysts were treated with the *P. harmala* extract for 24 h. The parasite viability was analyzed by PrestoBlue® reagent. The IC_{50} was analyzed by the Prism 5 software and represented as mean ± SD. The data obtained from 3 independent experiments.

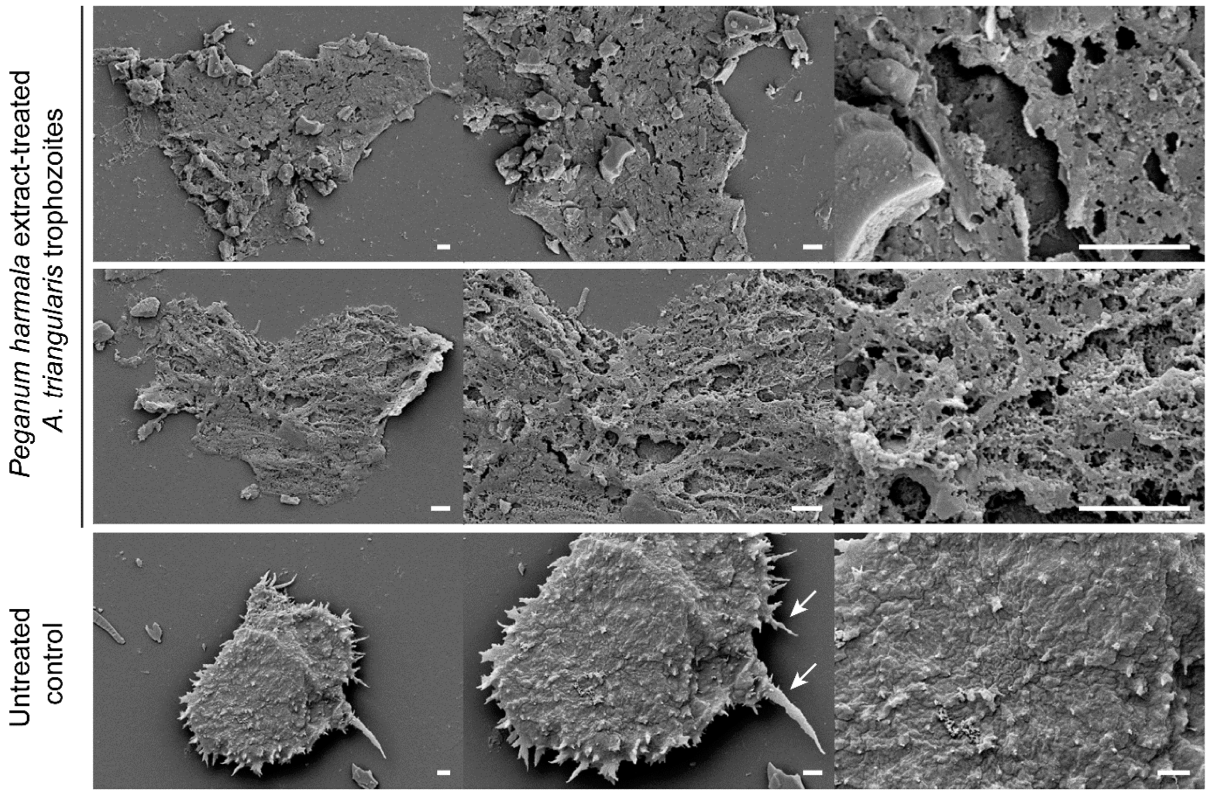

Figure S2. Scanning electron microscopy imaging of *A. triangularis* trophozoites treated with *P. harmala* extract. Cells were treated with 450 µg/mL (2xIC_{50}) of the extract for 24 h in a 24-well plate. Cells were then fixed and processed for SEM. Arrows indicate acanthopodia. Scale bars 1 µm.

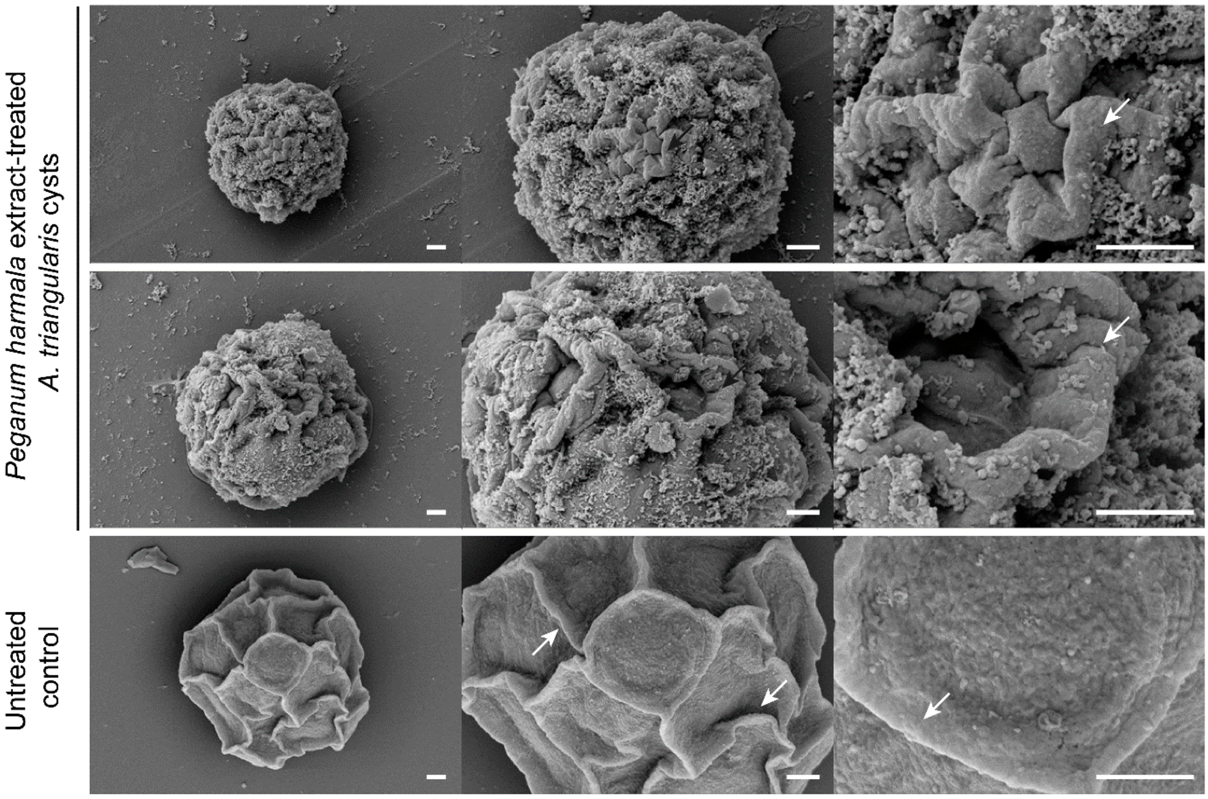

Figure S3. Scanning electron microscopy imaging of *A. triangularis* cysts treated with *P. harmala* extract. Cells were treated with 450 µg/mL (2xIC_{50}) of the extract for 24 h in a 24-well plate. Cells were then fixed and processed for SEM. Arrows indicate a pronounced edge. Scale bars 1 µm.

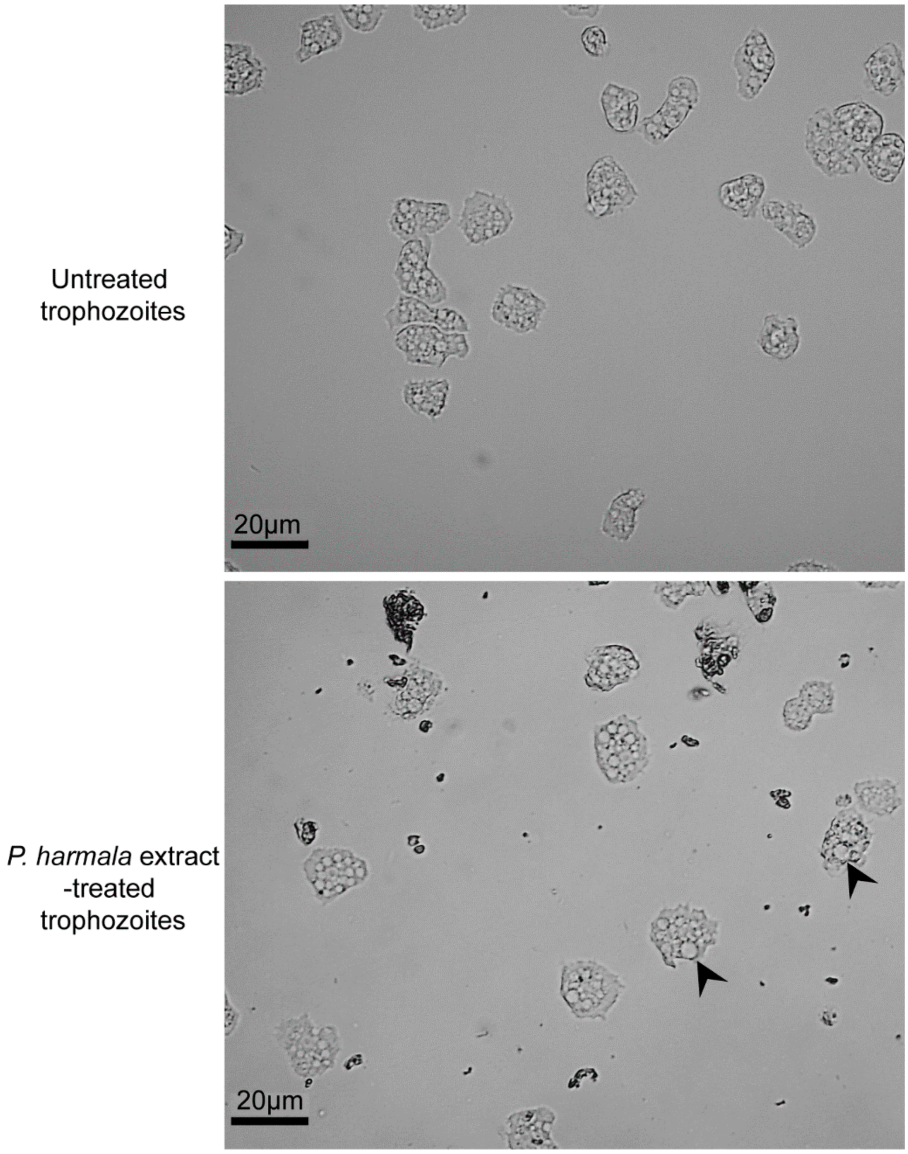

Figure S4. Representative images of vacuolization in surviving *A. triangularis* trophozoites upon *P. harmala* extract treatment. The parasites were treated with the extract at concentration of 225 μg/mL for 24 h. The surviving parasites containing vacuoles and/or enlarged vacuoles were analyzed. The enlarged vacuoles are indicated by black arrowhead. Scale bars 20 μm.

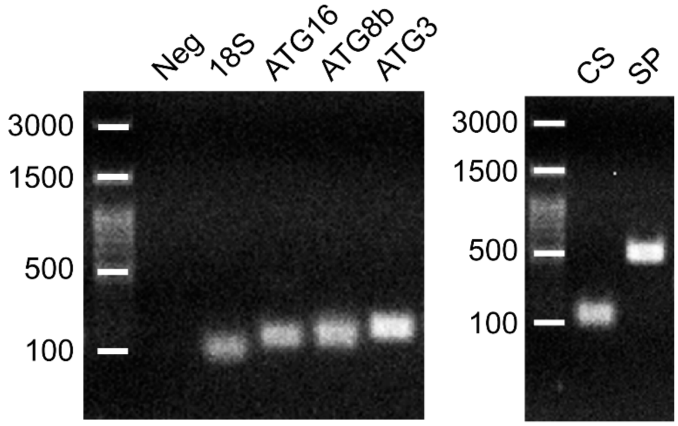

Figure S5. Gel electrophoresis of PCR product. Conventional PCR using primers specific to ATG genes, cellulose synthase (CS), serine proteinase (SP), including 18S rRNA was performed and the PCR product was run on 1.5% agarose gel. The first lane of each gel was a DNA ladder in bp. Neg is a negative control.

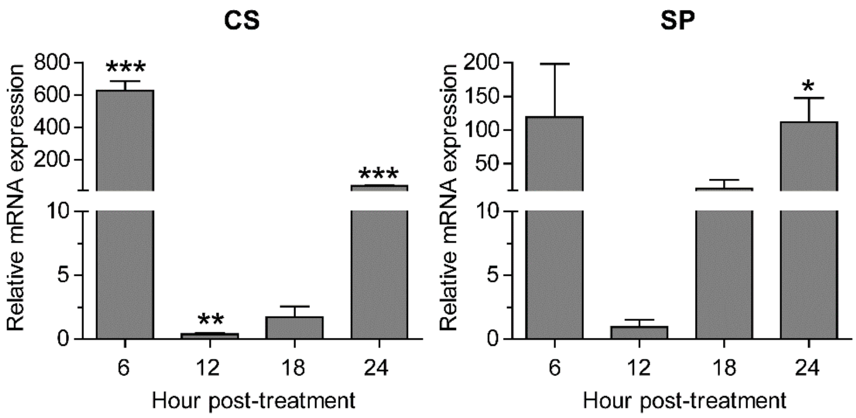

Figure S6. Transcriptional expression of other encystation-related genes. Expression level of cellulose synthase (CS) and serine proteinase (SP) mRNA was investigated. The cDNA samples were shared with autophagy-related genes analysis. 18S rRNA was used as internal normalization gene. The data were obtained from 3 independent experiments. Bar graphs showed mean ± SEM. *, $p < 0.05$; **, $p < 0.01$; ***, $p < 0.001$.

Table S1. Effect of *Peganum harmala* seed extract in combination with chlorhexidine against *Acanthamoeba triangularis* trophozoite.

***P. harmala* extract (µg/mL)**						
**512**	4.0±0	5.4±2.3	5.4±2.3	5.4±2.3	6.7±2.3	4.0±4.0
**256**	28.3±8.1	28.3±8.1	22.9±2.3	33.7±4.6	21.6±2.3	6.7±4.6
**128**	56.7±4.0	54.0±2.3	47.2±2.3	39.1±2.3	36.4±0	4.0±4.0
**64**	82.4±2.3	71.6±4.6	68.9±4.0	55.4±6.1	31.0±2.3	4.0±4.0
**32**	85.1±14.0	75.6±2.3	60.8±4.0	55.4±2.3	33.7±4.6	4.0±0
**0**	100.0±0	77.0±0	66.2±9.3	52.7±8.1	21.6±2.3	4.0±0
	**0**	**1**	**2**	**4**	**8**	**16**
			**Chlorhexidine (µg/mL)**			

Table S2. List of primers for quantitative PCR.

Gene	Genbank Accession No.	Forward (F)	Reverse (R)	*Acanthamoeba* spp.	References
ATG16	FJ906697	5’-AGCTTGACTTCCATCACGCTGA-3’	5’-TGTTTGAGGTTGGCCCGAA-3’	*A. castellanii*	(Song *et al.*, 2012)
ATG3	GU270859	5’-GCGCACGTACGATATCTCCATC-3’	5’-ATGAACACTTGGTTCGGCGTC-3’	*A. castellanii*	(Moon *et al.*, 2011)
ATG8b	KC524507.1	5’-CCGAGTTCCTGTGATCGTTGA-3’	5’-AGCTGTGTGACGGCAATATCG-3’	*A. castellanii*	(Moon *et al.*, 2013)
Cellulose synthase (CS)	EDCBI66TR	5’-TCATCTACATGTTCTGCGCCC-3’	5’-CGATCCAGTTGTTGAGCATGC-3’	*A. castellanii*	(Aqeel *et al.*, 2013; Moon *et al.*, 2014)
Serine proteinase (SP)	EU365404	5’-TCAAGGTGCTCGGATGCAAT-3’	5’-ATGTTAGCCACAGACTGCGTC-3’	*A. healyi*	(Moon *et al.*, 2008)
18S rRNA	-	5′-TCCAATTTTCTGCCACCGAA-3′	5′-ATCATTACCCTAGTCCTCGCGC-3′	*A. castellanii*	(Song *et al.*, 2012), (Moon *et al.*, 2008)

Table S3. *Acanthamoeba triangularis* DNA sequence by Sanger sequencing.

Target gene (Accession No.)	Primer	DNA sequence	Product length (bp).	% Identity[a]
**ATG16** (FJ906697)	F	5’-TCCGGGCCCTCTCCGGCTTACGCCCGCCACCTCACAGAACCCTTCAAAGCTAACGAGAGT CACGCCACACAGATGGAAGCTCGATCCTGTCCAACTCAAGAGACAGCAGCCTCAAGATGC TTGACATTCGGGCCAACCTCAAACAAA-3’	147	97.10
	R	5’-AAAAGTTGAGCTGCTGTCTCTTGAGTTGGACAGGATCGAGCTTCCATCTGTGTGGCGTGA CTCTCGTTAGCTTTGAAGGGTCTGTGAGGTGGCGGGCGTAGCGGAGAGGGGTACCCGGGC TCAGCGTGATGGAAGTCAAG-3’	140	-[b]
**ATG3** (GU270859)	F	5’-AAAATACAGAACGCCCAAGGTGTGGCTGTTTGGCTACGACGAGGTACACCCTGCTTTGAC CCCGTACCGCTCGGGTGGCCACCCGCAGACAAGGCAAAGCAACTGATTCTTCTTTGTGCC CTCTATTTCTATCCGCGGCGTGTAGAATGGCAACGGCCTGACGCCGAACCAAGTGTTCATA-3’	181	100.00
	R	5’-NTCTACGCCGCGGATAGAATAGAGGGCACAAAGAAGAATCAGTTGCTTTGCCTTGTCTGC GGGTGGCCACCCGAGCGGTACGGGGTCAAAGCAGGGTGTACCTCGTCGTAGCCAAACAGC CACACCTTGGGCGTCTGGTAGTACTTGTCGTAGGTGATGGAGATATCGTACGTGCGCA-3’	178	100.00
**ATG8b** (KC524507.1)	F	5’-CAGGGACAGCTCTTCCGACTTTCCAGAGGAGTACGCCTTGCGCCTTGCACCTATCCTCTC TATCCATGCTGCTGAAAGTTGCTCTTCGGTTCTCGGCTCCTCGTGGATATGCCCTCTCCA CCTATAGGC-3’	129	-[b]
	R	5’-CCCCCNACTGCGGAAGGAACTTTCTTCAGCATCTGGATATGGGATCTACGTGCAAGTGCA ACGCATACATTTTCTCTTAAATATCTCGAATAAGATCTCTCCCCTCGTGCTCTTCTCCAA TATCACGAGAACTCGGAC-3’	138	-[b]

[a] The DNA sequences were blasted against *A. castellanii* strain in NCBI database.
[b] No significant similarity found by NCBI-DNA blast (*A. castellanii* ATCC30011).

Table S3 (Cont.). *Acanthamoeba triangularis* DNA sequence by Sanger sequencing.

**Target gene** (Accession No.)	**Primer**	**DNA sequence**	**Product length (bp).**	**% Identity**[a]
**Cellulose synthase** (EDCBI66TR)	F	5’-ATCGCGAGGCGCCTGCCAGGCCAACGACCCGTTCAACACCAGCTCCTTCCTCTGGGTCTT CCTGCCCTACCTCTGCTTCCGCATGCTCAACAACTGGATCGA-3’	102	96.97
	R	5’-TCATCTACATGTTCTGCGCCCTGGTCTTCGTCTACTTCGGCGAGGCGCCCGCCAAGGCCA ACGACCCGTCAACACCAGCTCCTCCTCTGGTCTCTGCCATTAGTTGTACCGGG-3’	113	96.88
**Serine proteinase** (EU365404)	F	5’-GGANGAAGGGCTGTGCGCGGAGGCGATGAAGATGAAGATAATGATCAAGCTGCGCGGGTG ACGTACCAAGAGACGTAGGCGCAGAGAAAGCCTTCCTTGGTTCGCAGTATGCCCAGGATG TTCTCGATCTCGTCACCAAAGTCGAAGCCCACCTTGGCGTCGACTTGGGCGGCGGTGGTG GCGGGCGATGGTGCTTCACTCGTAGTCTTCGCGTCGGGTGCGGTACTCTTCCTAGGCCTC TTTGCCTTTGGGAAGAAGAAGACACGACATTCATAATAAATCCGTGAAAAAGAAGAAAAA GAAGAAGTGCGTTCCCCTCCAGTGGATGGAGCGGTTTACCTTTGGGCTACCGATGCCCTC GCTCGTGTCCTGCACGTCCATTTTATCTGTGTTCGGTTGGTTCAGAAGCTCGTCGTCATC GACGGTGGGCACCGAAGAAGCCGACGCAGTCTGTGGGCTAACATAAAC-3’	468	91.80
	R	5’-GTTTGGGCCCGTCGGATGACGACGAGCTTCTGAACCAACCGAACACAGATAAAATGGACG TGCAGGACACGAGCGAGGGCATCGGTAGCCCAAAGGTAAACCGCTCCATCCACTGGAGGG GAACGCACTTCTTCTTTTTCTTCTTTTTCACGGATTTATTATGAATGTCGTGTCTTCTTC TTCCCAAAGGCAAAGAGGCCTAGGAAGAGTACCGCACCCGACGCGAAGACTACGAGTGAA GCACCATCGCCCGCCACCACCGCCGCCCAAGTCGACGCCAAGGTGGGCTTCGACTTTGGT GACGAGATCGAGAACATCCTGGGCATACTGCGAACCAAGGAAGGCTTTCTCTGCGCCTAC GTCTCTTGGTACGTCACCCGCGCAGCTTGATCATTATCTTCATCTTCATCGCCTCCGCGC ACAGCCCTCATTCCTCATTCCTTATTTCATTGCATCCGAGCACCTTGAAA-3’	470	94.19
**18S rRNA**	F	5’-AATGGAATGGAATAGGACCTGTCCTCCTATTTTCAGTTGGTTTTGGCAGCGCGAGGACTA GGGTAATGATA-3’	71	98.48
	R	5’-TGAAATTAGGAAGGAACGGGTCCTATTCCATTATCCCATGCTAATGTATTCCGGTGGCAG AAAATTGGAAAATAGGAC-3’	78	92.75

[a] The DNA sequences were blasted against *A. castellanii* strain in NCBI database.
[b] No significant similarity found by NCBI-DNA blast (*A. castellanii* ATCC30011).
